# MiR-195 and Its Target SEMA6D Regulate Chemoresponse in Breast Cancer

**DOI:** 10.3390/cancers13235979

**Published:** 2021-11-28

**Authors:** Diana E. Baxter, Lisa M. Allinson, Waleed S. Al Amri, James A. Poulter, Arindam Pramanik, James L. Thorne, Eldo T. Verghese, Thomas A. Hughes

**Affiliations:** 1 School of Medicine, University of Leeds, Leeds LS9 7TF, UK; diana.baxter@cruk.manchester.ac.uk (D.E.B.); J.A.Poulter@leeds.ac.uk (J.A.P.); A.Pramanik@leeds.ac.uk (A.P.); 2 Cancer Research UK Manchester Institute, University of Manchester, Manchester SK10 4TG, UK; 3 Newcastle University Centre for Cancer, Faculty of Medical Sciences, Newcastle University, Newcastle upon Tyne NE2 4AD, UK; lisa.allinson@newcastle.ac.uk; 4 Department of Histopathology and Cytopathology, The Royal Hospital, Muscat, Oman; waleedsaid.alamri@moh.gov.om; 5 School of Food Science and Nutrition, University of Leeds, Leeds LS2 9JT, UK; J.L.Thorne@leeds.ac.uk; 6 Department of Histopathology, St. James’s University Hospital, Leeds LS9 7JX, UK; eldoverghese@nhs.net

**Keywords:** chemotherapy, neoadjuvant, microRNAs, RNA-seq, semaphorin

## Abstract

**Simple Summary:**

The resistance of cancer cells to cytotoxic chemotherapy limits cure rates in breast cancer, and a better understanding of resistance mechanisms will aid design of strategies to improve treatments. We have identified molecules that cause chemoresistance through comparison of matched breast cancer samples from before and after chemotherapy treatment, and by manipulating their levels in cultured breast cancer cells and measuring chemotherapy-induced cell death. We show that microRNA-195 and microRNA-26b induce resistance to chemotherapy in breast cancer by reducing the levels of the protein SEMA6D. Accordingly, levels of SEMA6D in breast cancers predict the survival of patients after chemotherapy. SEMA6D is a predictive marker and SEMA6D signaling presents a therapeutic opportunity for sensitizing cells to chemotherapy.

**Abstract:**

Background: poor prognosis primary breast cancers are typically treated with cytotoxic chemotherapy. However, recurrences remain relatively common even after this aggressive therapy. Comparison of matched tumours pre- and post-chemotherapy can allow identification of molecular characteristics of therapy resistance and thereby potentially aid discovery of novel predictive markers or targets for chemosensitisation. Through this comparison, we aimed to identify microRNAs associated with chemoresistance, define microRNA target genes, and assess targets as predictors of chemotherapy response. Methods: cancer cells were laser microdissected from matched breast cancer tissues pre- and post-chemotherapy from estrogen receptor positive/HER2 negative breast cancers showing partial responses to epirubicin/cyclophosphamide chemotherapy (*n* = 5). MicroRNA expression was profiled using qPCR arrays. MicroRNA/mRNA expression was manipulated in estrogen receptor positive/HER2 negative breast cancer cell lines (MCF7 and MDA-MB-175 cells) with mimics, inhibitors or siRNAs, and chemoresponse was assessed using MTT and colony forming survival assays. MicroRNA targets were identified by RNA-sequencing of microRNA mimic pull-downs, and comparison of these with mRNAs containing predicted microRNA binding sites. Survival correlations were tested using the METABRIC expression dataset (*n* = 1979). Results: miR-195 and miR-26b were consistently up-regulated after therapy, and changes in their expression in cell lines caused significant differences in chemotherapy sensitivity, in accordance with up-regulation driving resistance. SEMA6D was defined and confirmed as a target of the microRNAs. Reduced SEMA6D expression was significantly associated with chemoresistance, in accordance with SEMA6D being a down-stream effector of the microRNAs. Finally, low SEMA6D expression in breast cancers was significantly associated with poor survival after chemotherapy, but not after other therapies. Conclusions: microRNAs and their targets influence chemoresponse, allowing the identification of SEMA6D as a predictive marker for chemotherapy response that could be used to direct therapy or as a target in chemosensitisation strategies.

## 1. Introduction

Cytotoxic chemotherapy is a component of treatment for around a third of primary breast cancers, comprising those associated with relatively poor prognoses such as larger, lymph node positive, HER2-positive, or triple-negative tumours [1]. It is worth emphasizing that this treatment is therefore used in at least some cases of each of the classic molecular subtypes of breast cancer [2], including luminal subtypes (in the larger, high grade, node-positive or HER2 positive cases), the HER2-enriched subtype (in combination with HER2-targetting therapies), and the triple negatives (for which there are currently no alternative systemic treatments in the primary setting). Unfortunately, treatment failure in the form of cancer recurrences is relatively common in these groups, even after this aggressive therapy, and identification of chemotherapy resistance mechanisms is required to improve cancer outcomes [3]. Chemotherapy has traditionally been used in primary breast cancer in the adjuvant setting (after surgery). Alternatively, neoadjuvant (before surgery) chemotherapy is being increasingly used since it can down-stage tumours, thereby facilitating breast-conserving surgery in cases that would otherwise require mastectomies [4], and also allow opportunities for switching regimens if responses are poor, as assessed by longitudinal imaging [5]. From a research perspective, neoadjuvant chemotherapy provides the further advantage that matched tumour tissue from pre- and post-therapy can be available, allowing for the comparative identification of the therapy-associated changes within the cancer cells that may be associated with relative chemotherapy resistance. This strategy has been used successfully by our team and others, to identify chemoresponse-associated somatic mutations [6,7,8], and changes in protein [9,10] or mRNA expression [8,11]. MicroRNAs have received less attention in this context, with only a total of five previous studies following this design [12,13,14,15,16], leading to identification of only one microRNA, miR-18a, as a potential mediator of chemoresistance [13].

One key issue for these findings is that the cellular composition of breast cancer tissues changes dramatically after neoadjuvant therapy; post-therapy tissues are often fibrotic, with reduced representation of cancer cells [17,18] and reduced lymphocyte infiltration [19,20]. Therefore, the discovery of potential cancer cell autonomous resistance mechanisms through comparisons of whole tissue samples from pre- and post-therapy can be confounded by differences in tissue composition. This issue has previously been addressed by the exclusion of mRNAs for stromal-associated genes from any findings [11]. However, this approach is particularly problematic for microRNA analysis since data concerning which microRNAs could be regarded as stromal-specific are lacking. In order to circumvent this problem, we have previously performed laser microdissection to purify and enrich cancer cells from both matched samples, thereby allowing their more effective comparison [6,7]. We are aware of only one other study in which laser microdissection was carried out in the context of pre- and post-chemotherapy comparisons in breast cancer, in which mRNA expression profiles were compared before and after therapy [21], although it should be noted that only the post-therapy samples were laser microdissected.

Here, we have studied changes in expression of microRNAs after neoadjuvant chemotherapy in cancer cells, employing for the first-time laser microdissection to focus on the selection of potential resistance characteristics within these cells. For the discovery phase of our work, we have focused on estrogen receptor positive/HER2 negative cancers. This was because these cancers typically have a less than complete response to neoadjuvant chemotherapy [22], thereby increasing the cancer cell content of the post-therapy samples. Through this, we identified and then validate two microRNAs and a microRNA-target that confer chemoresistance in breast cancer cells, using cell lines representative of estrogen receptor positive/HER2 negative cancers. Subsequently, we assessed the impact of this microRNA target, SEMA6D, on patient outcomes, specifically after chemotherapy, in a cohort containing all the diversity of breast cancer subtypes that are treated with chemotherapy, demonstrating its applicability to this treatment modality beyond the estrogen receptor positive/HER2 negative subtype used for discovery.

## 2. Materials and Methods

### 2.1. Ethics, Patients and Tissue Samples

Ethical approval was obtained from Leeds (East) REC (ref 06/Q1206/180). Patients included were diagnosed within LTH NHS Trust (Leeds, UK) with estrogen receptor (ER) positive/HER2 negative primary invasive ductal breast carcinoma and were treated with 6 cycles of neoadjuvant epirubicin/cyclophosphamide, showing partial treatment resistance as determined by longitudinal MRI imaging and histopathology assessments of resections (partial treatment resistance was defined here as failing to achieve a complete pathological response, but evidence of some cell killing from reduction in tumour size on MRI scans). Patients were aged from 41 to 64 at diagnosis (mean 51) and tumours were grade 1 (1 case), 2 (2 cases) or 3 (2 cases); a table of clinico-pathological data on a patient-by-patient basis is shown in Table S1. Formalin-fixed, paraffin-embedded tumour tissue blocks were available representing matched diagnostic core biopsies (pre-therapy) and resections (post-therapy).

### 2.2. Laser Capture Microdissection, RNA Extraction and microRNA (miRNA) Profiling

Tumour tissue was sectioned and prepared, and epithelial cancer cells were isolated using a Zeiss/PALM machine (Zeiss; Oberkochen, Germany) or ArcturusXT System (ThermoFisher; Waltham, MA, USA) as previously described [7]. RNA was extracted from cells using AllPrep DNA/RNA FFPE Kits (Qiagen; Hilden, Germany) and quantified using a NanoDrop 2000 (ThermoFisher; Waltham, MA, USA). MicroRNAs were reverse transcribed (TaqMan MicroRNA Reverse Transcription Kit; Applied Biosystems; Carlsbad, CA, USA) and profiled (TaqMan MicroRNA Array A card v2; catalogue number 4398965, ThermoFisher, Waltham, MA, USA) following the manufacturer’s protocols. qPCR was performed on the 7900HT Fast Real-Time PCR System (Applied Biosystems; Carlsbad, CA, USA). Expression levels were normalized to the mean of all miRNAs expressed within that sample, and relative expression was calculated by comparing post-NAC with matched pre-NAC expression using the delta-delta Ct method [23].

### 2.3. Cell Culture and Transfections

MCF7 and MDA-MB-175 cells were purchased from ATCC (Manassas, VA, USA) and cultured in DMEM and Leibovitz’s L15 (Thermofisher; Waltham, MA, USA) respectively, supplemented with 10% FCS (Sigma; St Louis, MO, USA) and 1% penicillin/streptomycin (final concentrations 100 U/mL and 100 μg/mL) (Thermofisher; Waltham, MA, USA). Cells were grown at 37 °C in humidified 95% air/5% CO_2_ (MCF7) or 100% air (MDA-MB-175). Cell line identity was confirmed (STR profiles, Leeds Genomic Service, Leeds, UK) and cultures were consistently Mycoplasma negative (MycoAlert; Lonza; Basel, Switzerland). MiRNA mimics, hairpin inhibitors and scrambled controls were purchased from Dharmacon (Lafayette, LA, USA) and SEMA6D targeted (#76776117) and negative control siRNAs were purchased from IDT (Coralville, IA, USA). Cells were seeded into 96-well plates (MTT assays) or 24-well plates (clonogenic survival assays) and transfected at 70–80% confluency the next day. MiRNA mimic or inhibitor mixes were prepared in serum-free Opti-MEM, and transfection complexes prepared using Lipofectamine 2000 according to manufacturer’s protocols (Thermofisher; Waltham, MA, USA). Transfection complexes were added to cells in 2 volumes of Opti-MEM and one volume of standard medium. Medium was replaced with standard medium after 24 h. Biotinylated miRCURY LNA microRNA mimics or scrambled controls were used for the pulldown assays (Exiqon; Vedbæk, Denmark); MCF7 cells were transfected in T150 tissue culture flasks with Lipofectamine 2000 as described above. The epirubicin-resistant MCF7 line was developed by continuous culture in normal medium supplemented with epirubicin hydrochloride (Sigma; St Louis, MO, USA), initially at 1nM and then increasing over months to 350 nM.

### 2.4. Chemoresponse Assays

Cells were treated with 30nM-1μM epirubicin hydrochloride (Sigma; St Louis, MO, USA) for 24 h, starting 48 h post-transfection. For MTT assays, medium was removed and replaced with 25 µL of 5 mg/mL MTT (3-(4,5-Dimethylthiazol-2-yl)-2,5-Diphenyltetrazdium Bromide) (ThermoFisher; Waltham, MA, USA) and the cells were incubated under normal growth conditions in the dark. After 4 h, the MTT reagent was removed and the precipitates were dissolved in 50 µL 100% isopropanol. Absorbance was assessed at 570 nm using a Mithras LB940 plate reader (Berthold; Bad Wildbad, Germany). Clonogenic survival assays were performed as described previously [24]. In brief, cells were transfected, treated with epirubicin, and reseeded at low density (100 MCF7 or 200 MDA-MB-175 cells per well of 6-well plates) in technical duplicates without further epirubicin. MCF7 cells were reseeded in fresh media while MDA-MB-175 cells were seeded in conditioned media, prepared by being placed above the confluent MDA-MB-175 cells for 24 h and then centrifuged to ensure no carry-over of cells. Cells were incubated undisturbed under normal growth conditions for 14 days before colonies were stained with crystal violet (Sigma; St Louis, MO, USA). Colonies were counted macroscopically, regarding >30 cells as a colony. To validate colony counting reproducibility, 10 plates with varying numbers of colonies were counted independently by the authors DEB and LMA; the scores were highly correlated (R^2^ = 0.999).

### 2.5. Expression Analyses (qPCR, Western Blots)

For qPCR, total RNA was extracted using ReliaPrep RNA Cell Minipreps (Promega; Madison, WI, USA) according to the manufacturer’s protocols and quantified using a NanoDrop 2000 (Thermofisher; Waltham, MA, USA). For miRNA quantification, cDNA was synthesized using TaqMan Reverse Transcription Kits (Applied Biosystems; Carlsbad, CA, USA) and expression was quantified using TaqMan 2× Universal PCR Master Mix, No AmpErase UNG, and miRNA primers (Applied Biosystems; Carlsbad, CA, USA) according to the manufacturer’s protocols. For mRNA quantification, cDNA was synthesized using the GoScript Reverse Transcription System and expression was quantified using GoTaq qPCR Master Mix kits (Promega; Madison, WI, USA) according to the manufacturer’s protocols. Analyses were performed using the 7500 or QuantStudio 5 Real Time PCR Systems (Applied Biosystems; Carlsbad, CA, USA) in technical triplicates using standard modes and cycling conditions. The average Ct of The technical replicates was taken for each sample. Fold differences were calculated using the delta-delta Ct method [23], using RNU48 and ACTB as normalizers for the miRNA and mRNA respectively. For protein analyses, transfected cells were washed in PBS (4 °C) and then lysed (10 mM HEPES pH7.9, 10 mM KCl, 0.1 mM EDTA, 0.4% IGEPAL CA-630 (Sigma-Aldrich; St Louis, MO, USA), 1 mM DTT and Halt protease/phosphatase inhibitor (ThermoFisher; Waltham, MA, USA); 10 min; room temperature). Cells/buffer was centrifuged (15,000× *g*, 3 min, 4 °C) and the supernatants were collected. Proteins were quantified by Bradford assays (Merck; Kenilworth, NJ, USA). Samples were heated in Laemmli buffer (ThermoFisher; Waltham, MA, USA), 5 min at 90 °C, to denature proteins, and equal masses were loaded into each lane of 4–12% polyacrylamide gels (BioRad; Watford, UK). The proteins were separated, and then transferred to PVDF and blocked with 5% non-fat milk in Tris Buffered Saline, 0.1% Tween-20 (TBST) for 45 min. Membranes were incubated with a rabbit polyclonal antibody targeting the N-terminal region of SEMA6D (1:1200; ab198745; Abcam; Cambridge, UK) or rabbit monoclonal antibody against β-actin (1:2000 4970S; Cell Signalling Technologies; Beverly, MA, USA) in TBST/3% non-fat milk overnight (4 °C), and then with HRP-tagged secondary antibody (Cell Signalling Technologies; Beverly, MA, USA) (1:4000 in TBST, 3 h, room temperature). Blots were visualized using Pierce ECL reagents (ThermoFisher, Waltham, MA, USA) by ChemiDoc (BioRad; Watford, UK), and densitometry was performed using ImageJ (NIH Freeware, Bethesda, MD, USA).

### 2.6. Biotinylated miRNA Mimic Pulldowns, RNA-seq, and Data Analysis

Transfected MCF7 cells were harvested with trypsin-EDTA 24 h post-transfection, washed with PBS, collected by centrifugation (400× *g*, 5 min) and resuspended in ice-cold 1.5 mL lysis buffer (20 mM Tris pH7.5, 200 mM NaCl, 2.5 mM MgCl_2_, 0.05% Igepal, 1 mM PMSF, 1 mM DTT, 60U Superase-In RNase Inhibitor (ThermoFisher; Waltham, MA, USA)). Following cell lysis, samples were centrifuged (12,000 *g*, 15 min, 4 °C). Supernatants (excepting 50 µL aliquots that were kept as input samples) were added to pre-blocked Pierce High Capacity Streptavidin Agarose beads (ThermoFisher; Waltham, MA, USA) and incubated for 4 h at 4 °C whilst rotating. Beads were then washed with ice-cold lysis buffer. Total RNA was extracted using TRI-Reagent (Sigma; St Louis, MO, USA) following the manufacturer’s protocol. The aqueous phase was separated, and 5 µL GlycoBlue Coprecipitant (ThermoFisher; Waltham, MA, USA) was added before ethanol precipitation at −80 °C overnight. RNA was collected, washed, and resuspended in nuclease-free water. Total RNA samples were depleted of rRNA using Ribo-Zero Gold rRNA Removal Kits (Illumina; San Diego, CA, USA) according to the manufacturer’s instructions. Libraries were prepared using TruSeq Stranded Total RNA (Illumina; San Diego, CA, USA) reagents and protocols. Samples were quantified using Quanti-iTTM High Sensitivity dsDNA Assay Kits and the Qubit® 2.0 Fluorometer (Thermofisher; Waltham, MA, USA) and quality was assessed using the TapeStation 2200 and High Sensitivity D1000 ScreenTape (Agilent; Santa Clara, CA, USA). A total of 6 samples (pulldown: control, 195, 26b; input: control, 195, 26b) were sequenced in a single lane of paired-end sequencing using the HiSeq 3000 (Illumina; San Diego, CA, USA). Sequencing data (FASTQ) are available at the Sequence Read Archive (Available online: https://www.ncbi.nlm.nih.gov/sra) with the BioProject ID PRJNA725393. FASTQ files were aligned to the GRCh38 version of the human genome with STAR [25], using the two pass method that permits higher sensitivity of novel splice junction detection [26]. Header and read groups were then manually added to SAM files using Picard Tools (Broad Institute; Cambridge, MA, USA) to label individual samples. Data quality control was performed using FastQC (Babraham Institute; Cambridge, UK) before and after the removal of adapter sequences, primers and polyA tails using Cutadapt [27]; all samples passed quality control. Read counts were normalized to total reads per sample, and differential expression/pulldown was determined using DESeq (EMBL; Heidelberg, Germany).

### 2.7. Data Mining

The starBase resource (v2) [28,29] was available online: https://bio.tools/starbase and mRNAs with potential binding sites for miR-195 or miR-26b were identified using default settings. METABRIC data were accessed using cbioportal [30], as reported previously [31]. Records with SEMA6D expression data and suitable clinical annotation were identified (*n* = 1979; of these, *n* = 412 annotated as treated with cytotoxic chemotherapy for primary disease and *n* = 1567 annotated with sufficient treatment details to make clear cytotoxic chemotherapy was not used for primary disease; this does not include cases where treatment details were simply missing). A table summarizing the clinico-patholgical features of this cohort is shown as Table S2. Cases were dichotimised into low and high SEMA6D using arbitrary cut offs: ”low” was levels below −0.82 SD from the mean for the DFS analyses, or below −0.75 SD from the mean for the DSS analyses.

### 2.8. Statistical Analyses

Statistical analyses were performed using Prism (GraphPad, San Diego, CA, USA) using tests indicated in figure legends. *p* < 0.05 was taken to indicate significance.

## 3. Results

### 3.1. MiR-195 and miR-26b Expressions Were Consistently Changed Post-NAC

Our first aim was to identify miRNAs that were consistently differentially expressed in breast cancer cells between matched pre- and post-NAC samples, our hypothesis being that expression changes in cells surviving therapy are associated with relative therapy resistance. We focused on estrogen receptor positive/HER2 negative breast cancers treated with a specific chemotherapy regimen (epirubicin/cyclophosphamide) in order to reduce sample heterogeneity. This strategy also aided analysis of post-NAC cells, since estrogen receptor positive breast cancers are relatively resistant to chemotherapy as compared to other breast cancer subtypes [22], therefore increased numbers of post-therapy cells would be present.

We identified a cohort of 5 suitable cases demonstrating partial NAC resistance, and for which pre- and post-therapy tissues were available. Epithelial breast cancer cells were purified from tissues by laser capture microdissection to ensure that the changes identified were associated with differential expression in cancer cells, rather than potentially with changes in tissue composition, as are known to be induced by neoadjuvant therapy [32]. We have published representative images of this microdissection previously [6]. RNA was extracted from purified cells and the relative expression of 377 miRNAs was assessed using qPCR arrays. A total of 12 miRNAs were consistently differentially expressed between matched samples from all 5 patients; the fold differences for these are shown for all 5 patients, along with the mean fold change, in Table 1. A total of 10 miRNAs were up-regulated after treatment, while only 2 were down-regulated. We excluded 10 from further study by using the criterion of requiring an arbitrary minimum of 1.3-fold differential expression in every tumour, and subsequently focused on miR-195 and miR-26b, which were consistently up-regulated post-NAC by means of 2.6- and 4.9-fold respectively.

### 3.2. Altered Expression of miR-195 or miR-26b Controls Chemoresponse in ER Positive Breast Cancer Cell Lines

**Figure 1:**
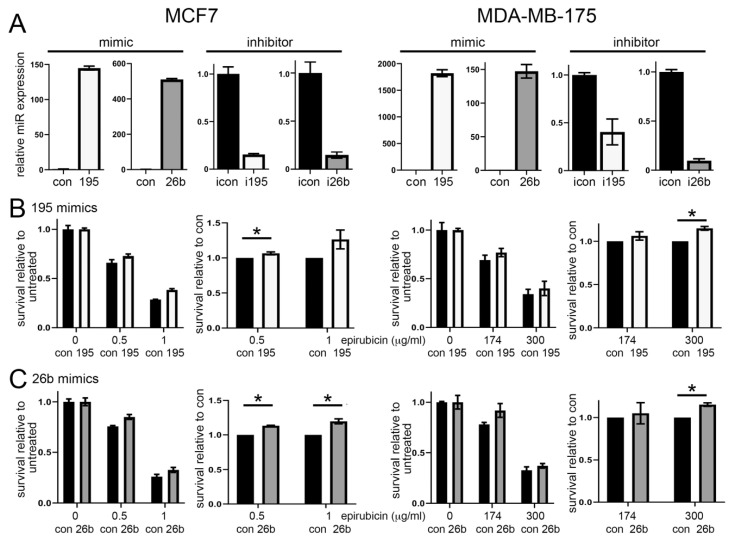
Increased miR-195 and miR-26b expression induces chemoresistance in estrogen receptor positive breast cancer cells. The MCF7 or MDA-MB-175 cells were transfected with miRNA mimics or mimic controls (195, 26b or con) or miRNA inhibitors or inhibitor control (i195, i26b or icon). (**A**) 48 h after transfection, qPCR was performed to assess the relative expression of miR-195 or miR-26b as appropriate. Data represent 1 biological experiment, with SD of technical triplicates. (**B**) and (**C**) 48 h after transfection, cells were treated with either two doses of epirubicin as indicated or with the vehicle control. Relative survival was determined using MTT assays 24 h later. Data are presented in two separate plots: left, relative to untreated (vehicle control), with 1 biological repeat showing error bars representing the SD of 3 replicate wells; right, relative to mimic controls, representative of means of 3 (MCF7) or 2 (MDA-MB-175) biological replicates, with error bars representing the SEM of biological replicates. Differences between the targeted mimic and mimic controls were tested using paired 1-tailed *t* tests; * indicates *p* < 0.05.

Next, we aimed to assess whether miR-195 or miR-26b were capable of directly modulating chemoresponse in breast cancer cells. Therefore, we transfected two separate breast cancer cell lines with either miRNA mimics to up-regulate expression, or miRNA inhibitors to down-regulate expression, and assessed the impact on chemosensitivity. We used MCF7 and MDA-MB-175 cells, since these are representative of the same breast cancer molecular subtype (estrogen receptor positive/HER2 negative) as our initial patient cohort. First, we transiently transfected each cell type with mimics of miR-195 or miR-26b, scrambled control mimics, or with miR-195 or miR-26b targeted or control miR inhibitors, and performed qPCR to assess the extents of up- or down-regulation (Figure 1A). We concluded that mimics allowed up-regulation by at least 144-fold, while inhibitors caused more modest down-regulation of 6- to 10-fold, although for miR-195 in the MDA-MB-175 cells, this was only 2.5-fold.

Having confirmed that we could experimentally manipulate levels of miR-195 and miR-26b, we next tested their impacts on chemoresponse to epirubicin, since this was the key cytotoxic agent with which the patients were treated, and is representative of the anthracycline family of chemotherapeutics, which are a component of the vast majority of chemotherapy regimens for primary breast cancer. Cells were transfected with mimics, inhibitors or controls as above, and were then treated with one of two doses of epirubicin, equating to ~IC25 and ~IC75 doses for each cell line, or with vehicle control. It was notable that MDA-MB-175 cells were intrinsically substantially more resistant to epirubicin than MCF7 cells, a fact we believe may relate to their lower growth rate. Cell survival was assessed using MTT assays. Survival is shown relative to the vehicle control in the left plots, while survival after transfection with the targeted mimic or inhibitor is shown relative to the scrambled control in the right plots (Figure 1B,C and Figure S1). The MiR-195 mimics induced significant increases in survival after treatment with the lower epirubicin dose in MCF7 cells (*p* < 0.05) and the higher dose in MDA-MB-175 (*p* = 0.05), with similar trends visible at the other doses (Figure 1B). The MiR-26b mimics induced significant increases in survival after treatment with both doses in MCF7 cells, and the higher dose in MDA-MB-175 cells (Figure 1C; *p* < 0.01). The inhibitors, however, had no significant influences on survival (Figure S1).

**Figure 2:**
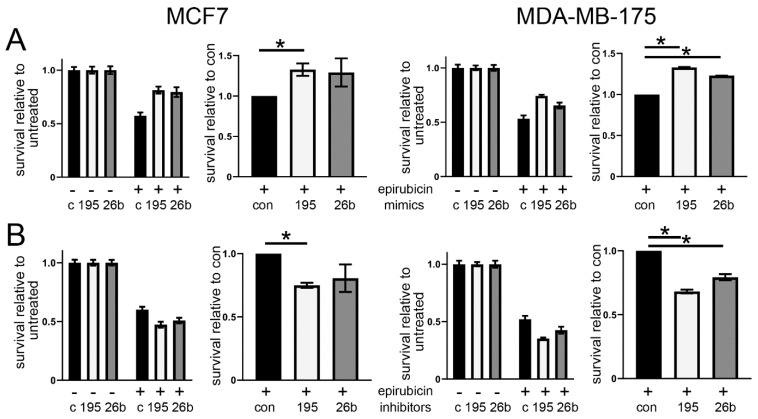
MiR-195 and miR-26b modify the chemoresponse of estrogen receptor positive breast cancer cells. The MCF7 or MDA-MB-175 cells were transfected with miRNA mimics or mimic controls (**A**) or miRNA inhibitors or inhibitor controls (**B**) (195, 26b or con (c)). 48 h after transfection, cells were treated with epirubicin (“+”; MCF7 30 nM; MDA-MB-175 600 nM) or with vehicle control (“−”). 24 h later, cells were replated at low density in fresh medium in order to determine relative survival in colony forming assays. Data are presented in two separate plots: left, relative to untreated (vehicle control), with 1 biological repeat showing error bars representing SD of 2 replicate wells; right, relative to mimic or inhibitor controls, representative of means of 3 (MCF7) or 2 (MDA-MB-175) biological replicates, with error bars representing the SEM of biological replicates. Differences between targeted mimic/inhibitor and controls were tested using paired 1-tailed *t* tests; * indicates *p* < 0.05.

In addition, we assessed epirubicin sensitivity after the manipulation of miRNA expression using clonogenic survival assays, in which cells were treated with the drug and then cultured at relatively low cell density in fresh medium without the drug for two weeks to allow a measurement of the proportions of cells that retained long-term proliferative ability. This assay is very sensitive to cellular damage that does not cause immediate short-term death, as detected by our MTT-based assay, but is nevertheless damage that compromises the replicative potential of the cells. Consequently, this assay is more reflective of some aspects of clinical cancer treatments where the key aim is to avoid cancer regrowth. Importantly, much lower drug doses were used in this clonogenic assay, reflecting the greater sensitivity to cellular damage. Cells were transfected as before, and then treated with the vehicle control or epirubicin at doses equating to ~IC50 for this assay, and clonogenic survival was determined; data are again presented relative to vehicle control (left) or relative to control mimics/inhibitors (right) (Figure 2). The MiR-195 mimics caused significant increases in survival in both cell lines (*p* < 0.05), while the miR-26b mimics showed similar effects, although significant in only the MDA-MB-175 cells (Figure 2A). By contrast with the MTT results, the miR-195 inhibitors caused significantly decreased survival in both cells (*p* < 0.02), as did the miR-26b inhibitors in the MDA-MB-175 cells (*p* < 0.03) (Figure 2B).

We also developed an epirubicin-resistant model system by continuous culture of MCF7 cells in increasing doses of epirubicin. The resistant cells (R) showed an IC50 of ~25µM as compared to ~2µM in the parental wildtype cells (WT) (Figure S2A). As a further test of the potential roles of miR-195 and miR-26b, we examined the expression of these miRNAs in both lines. We observed that miR-195 and miR-26b were up-regulated in the resistant line as compared to parental line (Figure S2B).

We concluded that miR-195 or miR-26b were regulators of response to epirubicin, with increased expression associated with relative resistance, as predicted from our assessment of patient samples (Table 1).

### 3.3. SEMA6D Is a Candidate Target of miR-195 and miR-26b

Having identified miR-195 and miR-26b as contributing to chemoresistance, our next aim was to identify target(s) of these miRNAs that contribute to this phenotype. Many methods have been reported for the identification of miRNA targets, however each is associated with a substantial false positive rate and there is no consensus for which is most effective [33]. In addition, miRNAs can target different genes in different cell types [34], a factor solely bioinformatics-based target prediction is unable to consider. We therefore combined methods, including both the most common computational prediction and a much more rarely performed biochemical pulldown approach, using our cell type of interest [35,36].

For computational predictions of miRNA targets, we used the on-line resource starBase [28,29], which gathers data from five commonly used predictive algorithms (miRanda, PicTar, TargetScan, RNA22 and PITA). A total of 55 and 24 genes were predicted as targets by all five algorithms for miR-195 and miR-26b respectively (Table S3). Next, we used an experimental approach; the MCF7 cells were transfected with biotin-tagged miR-195 or miR-26b mimics, or scrambled controls, with a view to using these mimics as baits to pull down mRNA targets for identification by RNA-seq. First, we checked that biotin tags did not interfere with miRNA function by confirming that tagged mimics continued to confer chemoresistance to MCF7 cells after transfection (Figure S3). Next, we transfected MCF7 cells, recovered tagged mimics on streptavidin beads, and sequenced RNA that was associated with these pulldowns. We identified mRNAs that were at least 100-fold over-represented in the miR-195 or miR-26b pulldowns as compared to the scrambled control pulldown, thereby defining transcripts representing 787 genes as potential targets of miR-195, and 787 genes (by chance, exactly the same number) as potential targets of miR-26b (Table S3).

Finally, we noted the genes that had been identified in both the computational predictions above and in our experimental pull-down as the strongest candidates: for miR-195, we were left with only one gene, SEMA6D, while for miR-26b we were left with none. We next tried a less stringent computational strategy by including genes predicted by only four of the five algorithms; in this case, the intersection between computational predictions and experimental candidates was SEMA6D and HOXA10 for miR-195, and SEMA6D (again) and HOXC4 for miR-26b. Therefore, we proceeded with SEMA6D as our key candidate downstream mediator of chemoresistance. The predicted binding sites for these miRNAs on the SEMA6D transcript are shown in Figure S4.

### 3.4. SEMA6D Is Targeted by miR-195 and Mediates Chemoresistance In Vitro

**Figure 3:**
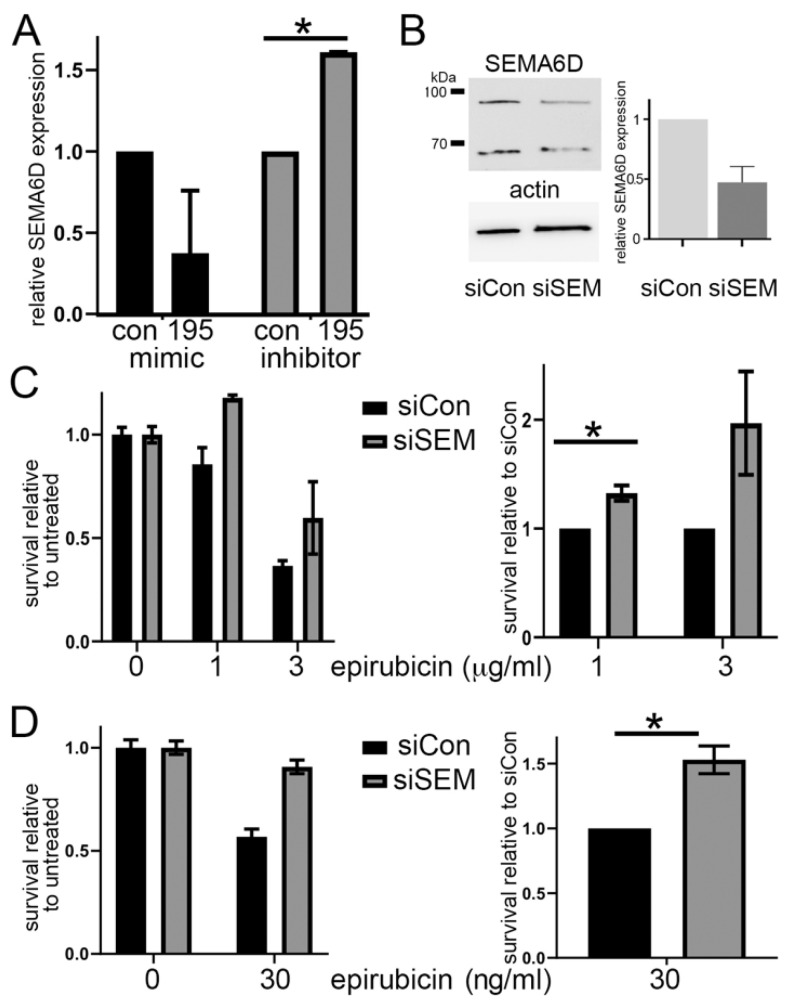
SEMA6D is a target of miR-195 and modifies chemoresponse. (**A**) The MCF7 cells were transfected with miR-195 mimics, inhibitors or controls. Relative SEMA6D expression was determined 72 h after transfection by qPCR. Data represent means (+/−SEM) of two biological repeats. Differences between targeted mimic/inhibitor and appropriate control were tested using paired 1-tailed *t* tests; * indicates *p* < 0.05. (**B**) The MCF7 cells were transfected with siRNA targeted against SEMA6D or the scrambled control. Proteins were extracted after 72 h and Western blots were performed for SEMA6D or actin (loading control). Relative SEMA6D expression was quantified using densitometry. Data represent 2 independent biological experiments (mean +/− SEM). (**C**,**D**) The MCF7 cells were transfected with siRNA targeted against SEMA6D or the scrambled control and treated with doses of epirubicin as indicated, or with vehicle control. Relative survival was determined using MTT (**C**) or colony forming assays (**D**). Data are presented in two separate plots: left, relative to untreated (vehicle control), with 1 biological repeat showing error bars representing the SD of replicate wells; right, relative to siRNA controls, representative of means of 2 (panel C) or 3 (panel D) biological replicates, with error bars representing the SEM of biological replicates. Differences between targeted siRNA and control were tested using paired 1-tailed *t* tests; * indicates *p* < 0.05.

Next, we assessed whether SEMA6D is a downstream target of our chemoresistance-related miRNA, focusing on miR-195 since SEMA6D was the only consistently predicted target for this miRNA. We transiently transfected MCF7 cells with miR-195 mimics or inhibitors, or controls, as before, and assessed the relative SEMA6D mRNA expression by qPCR (Figure 3A). In accordance with SEMA6D being a target of miR-195, expression of SEMA6D was significantly increased when miR-195 was inhibited (*p* < 0.0001), and showed a trend towards being decreased by miR-195 mimics. In support of this, SEMA6D expression was also decreased in our epirubicin-resistant MCF7 cell line (Figure S5), in which we had previously noted increased expression of miR-195. We concluded that SEMA6D is a target of miR-195, and that gene may mediate chemoresponse.

Our next aim was to test whether SEMA6D expression directly influenced chemoresponse in accordance with a function downstream of miR-195. We transiently transfected MCF7 cells with siRNAs targeting SEMA6D or a non-targeting control and confirmed that the siRNA effectively knocked-down SEMA6D expression. The SEMA6D was expressed as two specific protein species, as detected by an antibody recognizing the N-terminus; expression of both was reduced by the targeted siRNAs (Figure 3B). Then, we repeated these transfections and assessed sensitivity to epirubicin as previously, using either MTT assays (Figure 3C) or clonogenic survival assays (Figure 3D). Cells with reduced expression of SEMA6D were significantly more resistant to epirubicin both at the lower dose for the MTT assays (*p* < 0.05) and in the clonogenic survival assays (*p* < 0.01). We concluded that reduced SEMA6D expression caused relative epirubicin resistance, and therefore that miR-195 mediates chemoresponse, at least in part, through SEMA6D.

### 3.5. SEMA6D Is a Predictive Marker of Chemotherapy Response in Breast Cancer Patients

**Figure 4:**
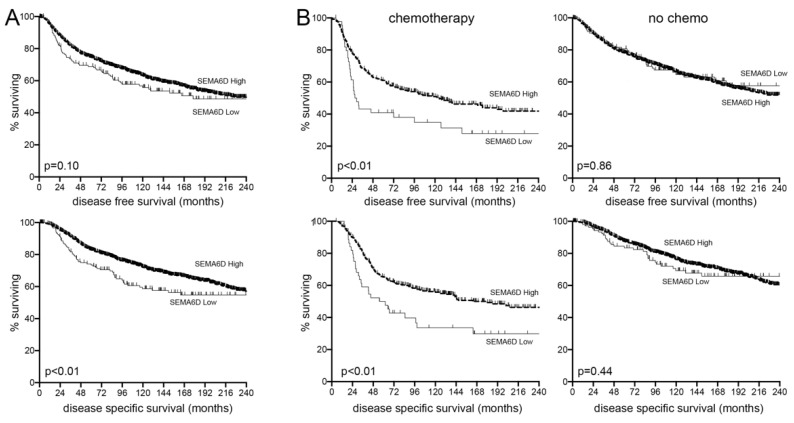
Low SEMA6D expression predicts poor survival in breast cancer patients specifically after chemotherapy. METABRIC transcriptomic data for breast cancers were accessed via cbioportal and records with SEMA6D expression data and suitable clinical annotation were identified (*n* = 1979). Kaplan–Meier survival analyses for disease-free survival (DFS; top plots) or disease–specific survival (DSS; bottom plots) were performed, comparing the relatively low and high SEMA6D expression groups within the full cohorts (**A**) or within those treated with chemotherapy (**B** left plots; *n* = 412) and those treated without (**B** right plots; *n* = 1567). Significance was assessed using Log Rank tests.

Having implicated SEMA6D expression in defining the chemoresponse of breast cancer cells, we next examined whether levels of SEMA6D would predict survival in breast cancer patients after chemotherapy. We accessed transcriptome and survival data for primary breast cancer cases from the METABRIC study [37], and tested whether SEMA6D expression levels correlated with either disease-free survival or disease-specific survival in these breast cancer cases (*n* = 1979; Figure 4A). Importantly, we also separated the cases into those who received chemotherapy for primary disease and those who did not (Figure 4B). We found that SEMA6D expression did not significantly impact disease-free survival in the complete cohort (Figure 4A top panel), although low expression was significantly correlated with disease specific survival (*p* = 0.005; Figure 4A bottom panel). Importantly, low expression significantly correlated with reduced disease-free and disease-specific survival in patients treated with chemotherapy (*p* = 0.009 and *p* = 0.004 respectively), but not in those treated without chemotherapy (Figure 4B). Furthermore, we also analysed whether receptor status impacted the predictive value of SEMA6D expression. Patients treated with chemotherapy were additionally subdivided into estrogen receptor positive or negative cases, and HER2 positive or negative cases, and separate Kaplan–Meier analyses were performed (Figure S6). Despite relatively low numbers of cases and/or events in these subgroups, significant predictive value for SEMA6D expression was maintained in the estrogen receptor negative subgroup (*p* = 0.017) and the HER2 positive subgroup (*p* = 0.022). We concluded that SEMA6D is a predictive marker of poor response, specifically to chemotherapy, with low expression allowing the identification of a group of patients prone to suffering very early recurrences after chemotherapy (inside 24 months).

## 4. Conclusions

We present the first examination of miRNA expression changes after clinical chemotherapy, specifically within breast cancer cells. We found only 12 miRNAs to show consistent expression changes, and fold changes were relatively small (<5-fold; Table 1 and Table S1). Previously, a much larger number of miRNAs have been identified as differentially expressed in breast tissues after chemotherapy; Lindholm et al. showed significant differences in 251 miRNAs [12], although this was based on differences in distributions of expression levels pre- and post-therapy, in contrast to our analysis that demanded consistent directions of differential expression between the matched samples in every case. As for the magnitude of differential expression, our data are typical, with other reports showing miRNAs to be differentially expressed mainly by 2- to 8-fold [12,14]. It should be noted that it is well-established that relatively small changes in miRNA expression can have substantial impacts on downstream functions [38]. Interestingly, miR-195 was previously reported as significantly up-regulated after chemotherapy in breast cancer, supporting our data, although our finding for miR-26b was not reproduced [12]. The critical difference between our study and previous reports is that we have focused on the cancer cells themselves, isolated by laser microdissection, thereby avoiding the confounding factor of chemotherapy-induced changes in the cellular constituents of tissues [17,19]. We believe this may explain the notable lack of consistency in published findings, represented by the facts that no single miRNA has had differential expression consistently demonstrated [12,13,14,15,16], and that only one miRNA identified in this way has previously been functionally validated successfully as a chemoresponse modulator (miR-18a [13], although its differential expression was not reproduced here or elsewhere [12]).

We have identified and validated two miRNAs, miR-195 and miR-26b, that functionally impact chemosensitivity in breast cancer cells (Figure 1 and Figure 2). MiR-195 has been studied extensively in breast cancer previously, mainly as a circulating biomarker for breast cancer diagnosis or prognosis [39,40]. Interestingly, higher circulating miR-195 was found to correlate with poor responses to neoadjuvant chemotherapy [40], which is concordant with our observation that higher cellular levels are associated with chemoresistance, although it is uncertain whether circulating miR-195 derives even in part from the cancer cells. This may, however, be a reasonable assumption, since miR-195 has been shown to be contained in breast cancer cell-derived extracellular vesicles, secretion of which increased on chemotherapy treatment, thereby inducing chemoresistant features in vitro and in mouse models [41]. More generally, miR-195 is thought to be a tumour suppressor in breast cancer, with lower expression in cancer as compared to normal tissues and ectopic expression associated with reduced proliferation and invasion [42]. MiR-26b is less well studied than miR-195 in the context of breast cancer, although, like miR-195, reports indicate down-regulation in cancers and an anti-proliferative role in over-expression [43,44]. A range of targets have been reported for either miR-195 or miR-26b in various contexts. However, since miRNA-mRNA pairings can differ depending on cell type [34], we wished to determine targets experimentally by using the cells in which we had noted their chemoresistance function. Therefore, we identified mRNAs bound to biotin-tagged miRNA mimics within cells using RNA-seq [36]. This technique is increasingly common, particularly in the context of assessing the lncRNA-miRNA interactions which are likely to be relatively stable and therefore experimentally more amenable [45], but has not previously been attempted for either miR-195 or miR-26b. To our surprise, through this and the prediction of miRNA binding sites, we identified the same shared target for both our miRNAs, namely semaphorin A6D (SEMA6D).

Dysregulation of semaphorins, a family of 20 related genes, is well established in cancer, and they are emerging as biomarkers and therapeutic targets [46], but relatively little is known specifically about SEMA6D in cancer. The family consists of trans-membrane proteins that act in cell-to-cell signalling through interactions with plexin receptors, working both very locally in full-length trans-membrane form or as secreted ligands when released through the proteolytic cleavage of ectodomains [46]. SEMA6D has been associated with a number of developmental roles, including cardiac morphogenesis [47], T-cell proliferation [48], and axonal guidance [49], functioning either as a ligand signaling through plexins (typically plexA1) or as a signal-transducing receptor itself. In the context of breast cancer, relatively high SEMA6D tissue expression has been associated with improved survival, particularly in the triple negative subtype that is invariably treated with chemotherapy [50]; this is compatible with our findings, although we fine-tune the observation to report improved survival as occurring only after chemotherapy (Figure 4). The other published finding concerning breast cancer is that the somatic SEMA6D mutations that were predicted to cause loss of gene function were identified in early onset breast cancers [51], a cancer type that is typically aggressive, frequently triple-negative, and associated with poor outcomes; this result, again, is potentially compatible with our finding that lower SEMA6D expression was associated with poorer outcomes. Some support is also available from other cancers; for example, in lung cancer, which is also invariably treated with cytotoxic chemotherapy, somatic SEMA6D mutations were found that reduced SEMA6D expression, and low SEMA6D expression was associated with poor outcomes [52]. In terms of mechanisms, it is challenging to speculate since it has not even been determined in these cells whether SEMA6D is working as a ligand for plexins, or as a receptor in its own right, therefore a multitude of potential down-stream signaling pathways exist [46]. However, there are potentially differences between different cancer types since higher SEMA6D expression was linked with chemotherapy resistance in osteosarcoma [53], albeit that this was resistance to cisplatin, which is a different class of chemotherapy agent. It should be noted that epirubicin (an anthracycline), the key chemotherapy agent in our study, acts by intercalating with DNA and also by inhibiting topoisomerase function [54], while the platinum-based agents, of which cisplatin is an example, act by inducing DNA base-adducts [55]. Nevertheless, our findings raise the prospect of attempting to manipulate semaphorin signalling to improve chemotherapy responses in breast cancer, and the potential of agonists for this pathway, or even using secreted semaphorins themselves as therapeutics has been explored previously in breast cancer [56] and other contexts [57], although not in combination with cytotoxic chemotherapy.

## Figures and Tables

**Table 1:** A total of 12 different miRNAs were consistently up-regulated or consistently down-regulated in breast cancer cells after neoadjuvant chemotherapy in 5 cases of estrogen receptor positive breast cancer. Cancer cells were isolated by laser capture microdissection from matched samples of pre- and post-neoadjuvant chemotherapy (NAC) breast cancer tissue. qPCR arrays were used to quantify the relative expression of 377 different microRNAs. Fold differences in post-NAC expression relative to pre-NAC are shown for each cancer case, along with the mean fold difference. * denotes fold differences that were estimated when the miR was not detected in one of the paired samples; the estimation is based on defining expression in this sample as the theoretical limit of detection (i.e., a cycle threshold of 40); these estimates were excluded from the assessment of mean fold differences.

MiRNA	Case 1	Case 2	Case 3	Case 4	Case 5	Mean
miR-195	3.46	1.84	1.70	1.33	4.50	2.57
miR-26b	3.39	1.54	1.92	1.79	15.7	4.87
let-7c	1.71	2.76	1.99	1.15	1.94	1.91
miR-10a	−2.33	−1.42	−14.4	−1.27	−1.36	−1.83
miR-26a	1.72	1.18	2.83	1.54	2.69	1.99
miR-330	1.70	1.32	1.11	1.82	>30 *	1.49
miR-335	3.15	4.94	1.16	1.55	>120 *	2.70
miR-362	1.68	1.03	1.27	2.85	>100 *	1.71
miR-483-5p	7020	3.34	1.13	2.27	19.9	1410
miR-885-5p	>870 *	1.14	42.0	2.69	>20 *	15.29
miR-625	27.8	1.24	1.10	4.04	>10 *	8.53
miR-365	−1.18	−1.47	−4.03	−1.19	−1.66	−1.55

## Data Availability

Data for this work are contained mainly within the article or supplementary material. Sequencing data (FASTQ) are available in a publicly accessible repository: the Sequence Read Archive (https://www.ncbi.nlm.nih.gov/sra) with the BioProject ID PRJNA725393.

## Supplementary Materials

The following are available online at https://www.mdpi.com/article/10.3390/cancers13235979/s1, Figure S1: Reduced miR-195 and miR-26b expression has little consistent impact on chemoresponse in estrogen receptor positive breast cancer cells as assessed by MTT assays. Figure S2: MiR-195 and miR-26b are overexpressed in a epirubicin-resistant cell line. Figure S3: Biotin-tagged miR-195 and miR-26b mimics induce chemoresistance in MCF7 cells. Figure S4: Predicted binding sites for miR-195 and miR-26b in the 3’ untranslated region of the SEMA6D transcript. Figure S5: SEMA6D expression was significantly reduced in epirubicin-resistant MCF7 cells as compared to parental. Figure S6: Low SEMA6D expression predicts poor survival in patients with estrogen receptor negative cancers and with HER2 positive cancers. Table S1: Clinico-pathological features of the discovery breast cancer cohort (n = 5; all estrogen receptor positive / HER2 negative). Table S2: Clinico-pathological features of the validation breast cancer cohort (n = 1979).
