# Supplementary material for: MiR-195 and Its Target SEMA6D Regulate Chemoresponse in Breast Cancer
Source: Cancers (Basel). 2021 Nov 28;13(23):5979. doi: 10.3390/cancers13235979 (PMC8656586; doi:10.3390/cancers13235979)
Supplement: Supplementary file 1 [file cancers-13-05979-s001.zip › cancers-1446649-supplementary.pdf]

Supplementary material

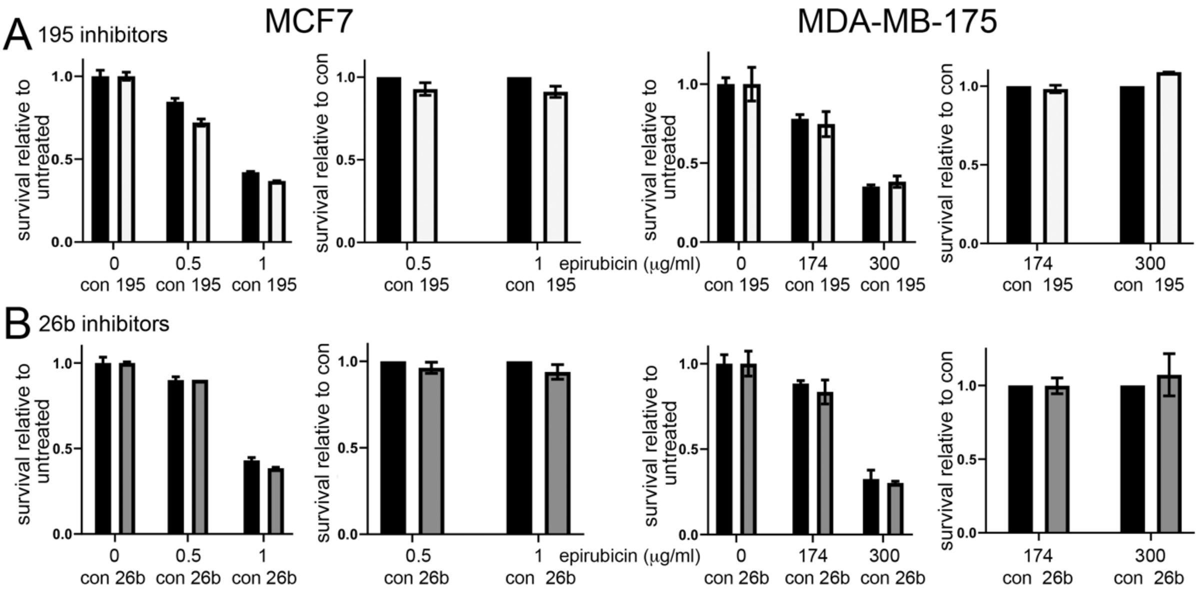

Figure S1. Reduced miR-195 and miR-26b expression has little consistent impact on chemoresponse in estrogen receptor positive breast cancer cells as assessed by MTT assays. MCF7 or MDA-MB-175 cells were transfected with miRNA inhibitors or inhibitor control (195 [panel A], 26b [panel B] or con). 48h after transfection, cells were treated with either of two doses of epirubicin as indicated or with vehicle control. Relative survival was determined using MTT assays 24h later. Data are presented in two separate plots: left, relative to untreated (vehicle control), with 1 biological repeat showing error bars representing SD of 3 replicate wells; right, relative to mimic controls, representative of means of 3 [MCF7] or 2 [MDA-MB-175] biological replicates, with error bars representing SEM of biological replicates. Differences between targeted mimic and mimic controls were tested using paired 1-tailed T tests; * indicates $p<0.05$.

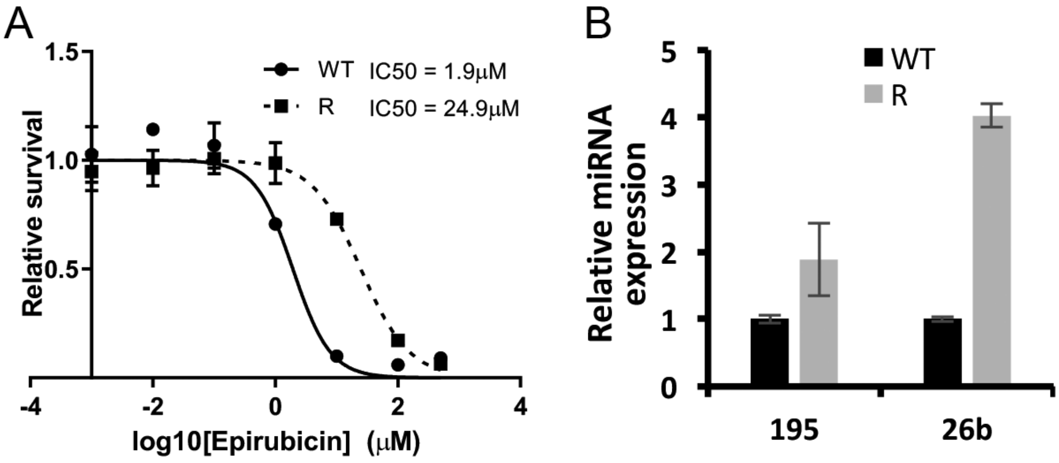

Figure S2. MiR-195 and miR-26b are overexpressed in a epirubicin-resistant cell line. Epirubicin-resistant MCF7 cells were developed by continuous culture in up to 350nM epirubicin. A) Relative survival of epirubicin-resistant MCF7 cells (R) and parental wild type MCF7 cells (WT) was assessed using MTT assays after treatment with a range of doses of epirubicin for 24h. Survival is shown relative to untreated and best fit sigmoidal response curves and IC50 values. Data represent means (+/- SD) of technical triplicates. B) Expression of miR-195 and miR-26b was determined in resistant (R) and wild type (WT) MCF7 cells using qPCR. Data represent means (+/- SD) of technical triplicates.

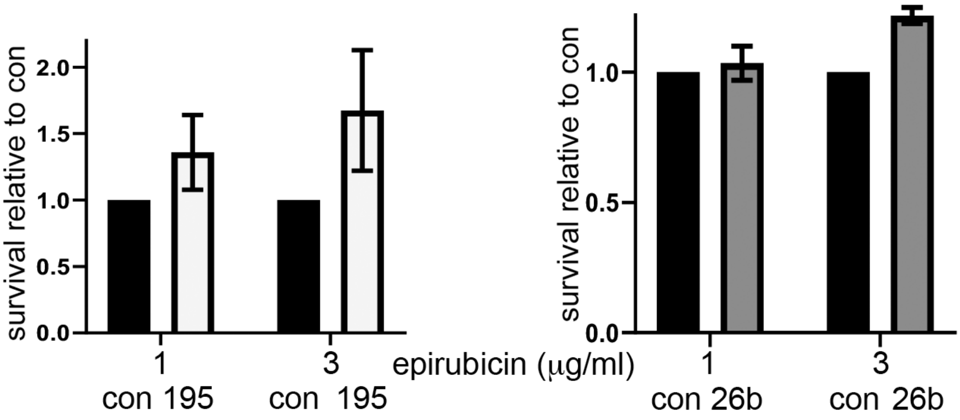

Figure S3. Biotin-tagged miR-195 and miR-26b mimics induce chemoresistance in MCF7 cells. MCF7 cells were transfected with biotin-tagged miRNA mimics or mimic controls (195, 26b or con) and 48h after transfection cells were treated with either of two doses of epirubicin as indicated or with vehicle control. Relative survival was determined using MTT assays 24h later. Data are presented relative to mimic controls, demonstrating the influence of miR-195 or miR-26b mimics on chemoresponse. Data are means of two biological replicates, with error bars representing SEM.

```
SEMA6D : 5' gcaAGUGAUUUUGUGCUGCUa 3'      chr15:48064811-48064818
               |:|: ||:|||||||||
miR-195: 3' cggUUAUAAAGACACGACGAu 5'


SEMA6D  : 5' auCUGUGAACAAAUACUUGAa 3'     chr15:48064304-48064310
               ||:|    || |||||||
miR-26b : 3' ugGAUAGGACUUAAUGAACUu 5'
```

Figure S4. Predicted binding sites for miR-195 and miR-26b in the 3’ untranslated region of the SEMA6D transcript.

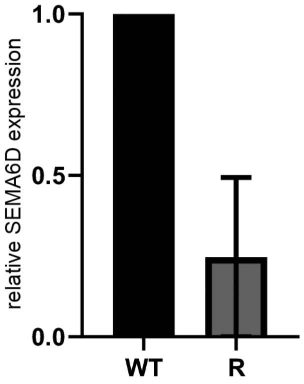

Figure S5. SEMA6D expression was significantly reduced in epirubicin-resistant MCF7 cells as compared to parental. Epirubicin-resistant MCF7 cells were developed by continuous culture in up to 350nM epirubicin. Expression of SEMA6D was determined in resistant (R) and wild type (WT) cells using qPCR. Data represent means (+/- SEM) of 2 biological replicates.

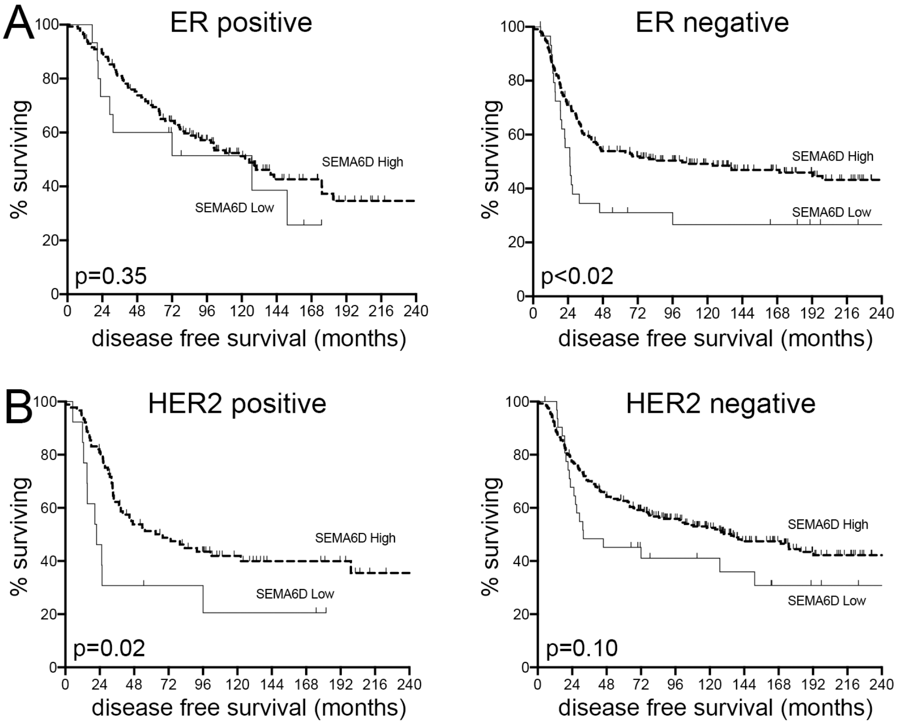

Figure S6. Low SEMA6D expression predicts poor survival in patients with estrogen receptor negative cancers and with HER2 positive cancers. METABRIC transcriptomic data for breast cancers were accessed via cbioportal and records for cases treated with cytotoxic chemotherapy for primary disease, and with SEMA6D expression data and suitable clinical annotation were identified (n = 412). Kaplan-Meier survival analyses for disease free survival (DFS) comparing relatively low and high SEMA6D expression groups were performed in tumours that were: A) estrogen receptor positive (n = 158) or negative (n = 254), or B) HER2 receptor positive (n = 103) or negative (n = 309). Significance was assessed using Log Rank tests (p values are shown).

Patient	Age	Histology	Nodal status	Grade
**1**	50	IDC	positive	3
**2**	41	IDC	positive	3
**3**	52	IDC	positive	2
**4**	51	Invasive mixed ductal and lobular	positive	1
**5**	63	IDC	positive	2

Table S1. Clinico-pathological features of the discovery breast cancer cohort (n=5; all estrogen receptor positive / HER2 negative). IDC, invasive ductal carcinoma

	All patients	Chemotherapy	No chemotherapy
Patients	1979	412	1567
Median age at diagnosis (range)	62 (22-96)	50.4 (22-86)	65 (26-96)
Median tumour size (range)	23 (1-182)	26 (1-182)	21.8 (1-150)
Subtype (by PAM50)			
Luminal A	699	56	643
Luminal B	475	47	428
Her2 positive	224	88	136
Basal-like	209	113	96
Claudin-low	218	73	145
Other/not applicable	154	36	119
Histological Grade			
1	169	8	161
2	771	76	695
3	951	323	628
Data not available	88	5	83
Tumour Stage			
0	12	0	12
1	500	20	480
2	825	234	591
3	118	60	58
4	10	3	7
Data not available	514	95	419
Disease free survival median follow-up (months)	100.7	70.3	107.6
Disease-specific survival median follow-up (months)	116.4	84.3	122.8

Table S2. Clinico-pathological features of the validation breast cancer cohort (n=1979).

excelsheet

Table S3. Genes with transcripts predicted to be miR-195 or miR-26b targets by all 5 predictive algorithms included in starBase, or experimentally identified as targets by pulldown assay.
